# Supplementary material for: Iterative improvement in the automatic modular design of robot swarms
Source: PeerJ Comput Sci. 2020 Dec 7;6:e322. doi: 10.7717/peerj-cs.322 (PMC7924708; doi:10.7717/peerj-cs.322)
Supplement: Supplemental Information 3 [file peerj-cs-06-322-s003.zip › argos3/doc/api/standalone/a00462.html]

ARGoS: plugins/robots/foot-bot/simulator/footbot\_distance\_scanner\_rotzonly\_sensor.cpp File Reference


- Main Page
- Related Pages
- Namespaces
- Classes
- Files

- File List
- File Members

# plugins/robots/foot-bot/simulator/footbot\_distance\_scanner\_rotzonly\_sensor.cpp File Reference

`#include "footbot_distance_scanner_rotzonly_sensor.h"`  
`#include <argos3/core/simulator/entity/composable_entity.h>`  
`#include <argos3/core/simulator/entity/controllable_entity.h>`  
`#include <argos3/core/simulator/simulator.h>`  
`#include <argos3/core/simulator/space/space.h>`  

Include dependency graph for footbot\_distance\_scanner\_rotzonly\_sensor.cpp:

Go to the source code of this file.

|  |  |
| --- | --- |
| Namespaces | |
| namespace | argos |

|  |  |
| --- | --- |
|  | The namespace containing all the ARGoS related code. |

| Defines | |
| #define | ADD\_READING(RAYS, MAP, INDEX, MINDIST) |
| #define | ADD\_READINGS(RAYS, MAP, MINDIST) |
| #define | CALCULATE\_SHORT\_RANGE\_RAY(ANGLE, INDEX) |
| #define | CALCULATE\_LONG\_RANGE\_RAY(ANGLE, INDEX) |
| Functions | |
|  | argos::REGISTER\_SENSOR (CFootBotDistanceScannerRotZOnlySensor,"footbot\_distance\_scanner","rot\_z\_only","Carlo Pinciroli [ilpincy@gmail.com]","1.0","The foot-bot distance scanner sensor (optimized for 2D).","This sensor accesses the foot-bot distance scanner sensor. For a complete\n""description of its usage, refer to the common interface.\n""In this implementation, the readings are calculated under the assumption that\n""the foot-bot is always parallel to the XY plane, i.e., it rotates only around\n""the Z axis. This implementation is faster than a 3D one and should be used\n""only when the assumption about the foot-bot rotation holds.\n\n""REQUIRED XML CONFIGURATION\n\n"" <controllers>\n"" ...\n"" <my\_controller ...>\n"" ...\n"" <sensors>\n"" ...\n"" <footbot\_distance\_scanner implementation=\"rot\_z\_only\" />\n"" ...\n"" </sensors>\n"" ...\n"" </my\_controller>\n"" ...\n"" </controllers>\n\n""OPTIONAL XML CONFIGURATION\n\n""It is possible to draw the rays shot by the distance scanner in the OpenGL\n""visualization. This can be useful for sensor debugging but also to understand\n""what's wrong in your controller. In OpenGL, the rays are drawn in cyan when\n""they are not obstructed and in purple when they are. In case a ray is\n""obstructed, a black dot is drawn where the intersection occurred.\n""To turn this functionality on, add the attribute 'show\_rays=\"true\"' in the\n""XML as in this example:\n\n"" <controllers>\n"" ...\n"" <my\_controller ...>\n"" ...\n"" <sensors>\n"" ...\n"" <footbot\_distance\_scanner implementation=\"rot\_z\_only\"\n"" show\_rays=\"true\" />\n"" ...\n"" </sensors>\n"" ...\n"" </my\_controller>\n"" ...\n"" </controllers>\n","Usable") |

---

## Define Documentation

|  |  |  |  |  |  |
| --- | --- | --- | --- | --- | --- |
| #define ADD\_READING | ( | RAYS, |  |  |  |
|  |  | MAP, |  |  |  |
|  |  | INDEX, |  |  |  |
|  |  | MINDIST |  | ) |  |

**Value:**

```
cAngle += cInterSensorSpan;                              \
   cAngle.SignedNormalize();                                \
   fReading = CalculateReadingForRay(RAYS[INDEX],MINDIST);  \
   MAP[cAngle] = fReading;                                  \
   m_tReadingsMap[cAngle] = fReading;
```

Definition at line 146 of file footbot\_distance\_scanner\_rotzonly\_sensor.cpp.

|  |  |  |  |  |  |
| --- | --- | --- | --- | --- | --- |
| #define ADD\_READINGS | ( | RAYS, |  |  |  |
|  |  | MAP, |  |  |  |
|  |  | MINDIST |  | ) |  |

**Value:**

```
ADD_READING(RAYS,MAP,1,MINDIST)              \
   ADD_READING(RAYS,MAP,2,MINDIST)              \
   ADD_READING(RAYS,MAP,3,MINDIST)              \
   ADD_READING(RAYS,MAP,4,MINDIST)              \
   ADD_READING(RAYS,MAP,5,MINDIST)
```

Definition at line 153 of file footbot\_distance\_scanner\_rotzonly\_sensor.cpp.

|  |  |  |  |  |  |
| --- | --- | --- | --- | --- | --- |
| #define CALCULATE\_LONG\_RANGE\_RAY | ( | ANGLE, |  |  |  |
|  |  | INDEX |  | ) |  |

**Value:**

```
m_cDirection.RotateZ(ANGLE);                             \
   m_cOriginRayStart = m_cDirection;                        \
   m_cOriginRayEnd = m_cDirection;                          \
   m_cOriginRayStart *= LONG_RANGE_RAY_START;               \
   m_cOriginRayEnd *= LONG_RANGE_RAY_END;                   \
   m_cRayStart = m_pcEmbodiedEntity->GetOriginAnchor().Position;  \
   m_cRayStart += m_cOriginRayStart;                        \
   m_cRayStart.SetZ(m_cRayStart.GetZ() + SENSOR_ELEVATION); \
   m_cRayEnd = m_pcEmbodiedEntity->GetOriginAnchor().Position;  \
   m_cRayEnd += m_cOriginRayEnd;                            \
   m_cRayEnd.SetZ(m_cRayEnd.GetZ() + SENSOR_ELEVATION);     \
   m_cLongRangeRays1[INDEX].Set(m_cRayStart, m_cRayEnd);    \
   m_cRayStart = m_pcEmbodiedEntity->GetOriginAnchor().Position;  \
   m_cRayStart -= m_cOriginRayStart;                        \
   m_cRayStart.SetZ(m_cRayStart.GetZ() + SENSOR_ELEVATION); \
   m_cRayEnd = m_pcEmbodiedEntity->GetOriginAnchor().Position;  \
   m_cRayEnd -= m_cOriginRayEnd;                            \
   m_cRayEnd.SetZ(m_cRayEnd.GetZ() + SENSOR_ELEVATION);     \
   m_cLongRangeRays3[INDEX].Set(m_cRayStart, m_cRayEnd);
```

Definition at line 250 of file footbot\_distance\_scanner\_rotzonly\_sensor.cpp.

|  |  |  |  |  |  |
| --- | --- | --- | --- | --- | --- |
| #define CALCULATE\_SHORT\_RANGE\_RAY | ( | ANGLE, |  |  |  |
|  |  | INDEX |  | ) |  |

**Value:**

```
m_cDirection.RotateZ(ANGLE);                             \
   m_cOriginRayStart = m_cDirection;                        \
   m_cOriginRayEnd = m_cDirection;                          \
   m_cOriginRayStart *= SHORT_RANGE_RAY_START;              \
   m_cOriginRayEnd *= SHORT_RANGE_RAY_END;                  \
   m_cRayStart = m_pcEmbodiedEntity->GetOriginAnchor().Position;  \
   m_cRayStart += m_cOriginRayStart;                        \
   m_cRayStart.SetZ(m_cRayStart.GetZ() + SENSOR_ELEVATION); \
   m_cRayEnd = m_pcEmbodiedEntity->GetOriginAnchor().Position;  \
   m_cRayEnd += m_cOriginRayEnd;                            \
   m_cRayEnd.SetZ(m_cRayEnd.GetZ() + SENSOR_ELEVATION);     \
   m_cShortRangeRays0[INDEX].Set(m_cRayStart, m_cRayEnd);   \
   m_cRayStart = m_pcEmbodiedEntity->GetOriginAnchor().Position;  \
   m_cRayStart -= m_cOriginRayStart;                        \
   m_cRayStart.SetZ(m_cRayStart.GetZ() + SENSOR_ELEVATION); \
   m_cRayEnd = m_pcEmbodiedEntity->GetOriginAnchor().Position;  \
   m_cRayEnd -= m_cOriginRayEnd;                            \
   m_cRayEnd.SetZ(m_cRayEnd.GetZ() + SENSOR_ELEVATION);     \
   m_cShortRangeRays2[INDEX].Set(m_cRayStart, m_cRayEnd);   \
                                                            \
```

Definition at line 228 of file footbot\_distance\_scanner\_rotzonly\_sensor.cpp.

---

Generated on 10 Jul 2018 for ARGoS by 
 1.6.1
